# Phylogenetic inference in Rafflesiales: the influence of rate heterogeneity and horizontal gene transfer

**DOI:** 10.1186/1471-2148-4-40

**Published:** 2004-10-20

**Authors:** Daniel L Nickrent, Albert Blarer, Yin-Long Qiu, Romina Vidal-Russell, Frank E Anderson

**Affiliations:** 1 Department of Plant Biology, Southern Illinois University, Carbondale, IL 62901-6509, USA; 2 Institute of Systematic Botany, University of Zurich, 8008 Zurich, Switzerland; 3 Department of Ecology and Evolutionary Biology, University of Michigan, Ann Arbor, MI 48109-1048, USA; 4 Department of Zoology, Southern Illinois University, Carbondale IL, 62901-6501, USA

## Abstract

**Background:**

The phylogenetic relationships among the holoparasites of Rafflesiales have remained enigmatic for over a century. Recent molecular phylogenetic studies using the mitochondrial *matR *gene placed *Rafflesia*, *Rhizanthes *and *Sapria *(Rafflesiaceae s. str.) in the angiosperm order Malpighiales and *Mitrastema *(Mitrastemonaceae) in Ericales. These phylogenetic studies did not, however, sample two additional groups traditionally classified within Rafflesiales (Apodantheaceae and Cytinaceae). Here we provide molecular phylogenetic evidence using DNA sequence data from mitochondrial and nuclear genes for representatives of all genera in Rafflesiales.

**Results:**

Our analyses indicate that the phylogenetic affinities of the large-flowered clade and *Mitrastema*, ascertained using mitochondrial *matR*, are congruent with results from nuclear SSU rDNA when these data are analyzed using maximum likelihood and Bayesian methods. The relationship of Cytinaceae to Malvales was recovered in all analyses. Relationships between Apodanthaceae and photosynthetic angiosperms varied depending upon the data partition: Malvales (3-gene), Cucurbitales (*matR*) or Fabales (*atp1*). The latter incongruencies suggest that horizontal gene transfer (HGT) may be affecting the mitochondrial gene topologies. The lack of association between *Mitrastema *and Ericales using *atp1 *is suggestive of HGT, but greater sampling within eudicots is needed to test this hypothesis further.

**Conclusions:**

Rafflesiales are not monophyletic but composed of three or four independent lineages (families): Rafflesiaceae, Mitrastemonaceae, Apodanthaceae and Cytinaceae. Long-branch attraction appears to be misleading parsimony analyses of nuclear small-subunit rDNA data, but model-based methods (maximum likelihood and Bayesian analyses) recover a topology that is congruent with the mitochondrial *matR *gene tree, thus providing compelling evidence for organismal relationships. Horizontal gene transfer appears to be influencing only some taxa and some mitochondrial genes, thus indicating that the process is acting at the single gene (not whole genome) level.

## Background

Combining gene sequences from multiple subcellular compartments continues to provide increasingly well-resolved flowering plant phylogenies [1] and these have precipitated a new classification for angiosperms [2]. Whereas most groups have been placed at the ordinal level, seven of the 18 "taxa of uncertain position" are holoparasitic, nonphotosynthetic flowering plants. These parasites have been difficult to ally with green plants owing to extreme reduction and/or loss of morphological features [3]. Chloroplast genes commonly used to infer land plant phylogenetic relationships either show elevated substitution rates or are absent in these holoparasites [3-5]. Moreover, nuclear ribosomal genes also show greatly increased rates [6], thus analytical methods that accommodate such among-lineage rate heterogeneity must be used.

Rafflesiales are a fascinating and enigmatic group of holoparasitic plants that includes *Rafflesia*, whose meter-wide flowers are the largest among all angiosperms, and *Pilostyles*, whose flowers are less than a centimeter in diameter. Such wide morphological variation has resulted in classifications that comprise four families: 1) the "small-flowered clade" (Apodanthaceae) with *Apodanthes*, *Berlinianche*, and *Pilostyles*, 2) the "large-flowered clade" (Rafflesiaceae s. str.) with *Rafflesia*, *Rhizanthes*, and *Sapria*, 3) the "inflorescence clade" (Cytinaceae) with *Bdallophyton *and *Cytinus*, and 4) the "hypogynous clade" (Mitrastemonaceae) with *Mitrastema *[7,8].

Recently, Barkman et al. [9] used DNA sequences of the mitochondrial gene *matR *to identify the closest photosynthetic relatives of two clades within Rafflesiales. Three genera, representing two of the four families in the order, were used in that study: *Rafflesia *and *Rhizanthes *(Rafflesiaceae s. str.) and *Mitrastema *(Mitrastemonaceae). Analyses of the *matR *data placed Rafflesiaceae s. str. within Malpighiales, an order that includes passionflowers (*Passiflora*), willow (*Salix*), and violet (*Viola*). Mitrastemonaceae was placed within Ericales, an order containing blueberries (*Vaccinium*), primroses (*Primula*), and tea (*Camellia*). The authors argued that these results were robust because they were congruent using different analytical methods (parsimony, neighbor-joining, Bayesian) and were not affected by long-branch attraction artifacts [10]. Moreover, because sequences from host plant lineages were included, and the parasites did not emerge as sister to these lineages, contamination and horizontal gene transfer (HGT) were discounted.

In this study we expand upon the previous analysis [9] by including representatives of all Rafflesiales genera and families, thus allowing us to address the question of monophyly of the order. Moreover, parsimony, likelihood and Bayesian analyses were conducted on genes derived from all three subcellular compartments. These results were compared to assess the impact of artifacts such as long-branch attraction and HGT on various relationships. The data sets used were 1) mitochondrial *matR*, 2) mitochondrial *atp1 *and 3) a "3-gene" data set consisting of nuclear SSU rDNA plus two chloroplast genes: *rbcL *and *atpB *(the latter two only from nonparasites).

## Results

**Figure 1:**
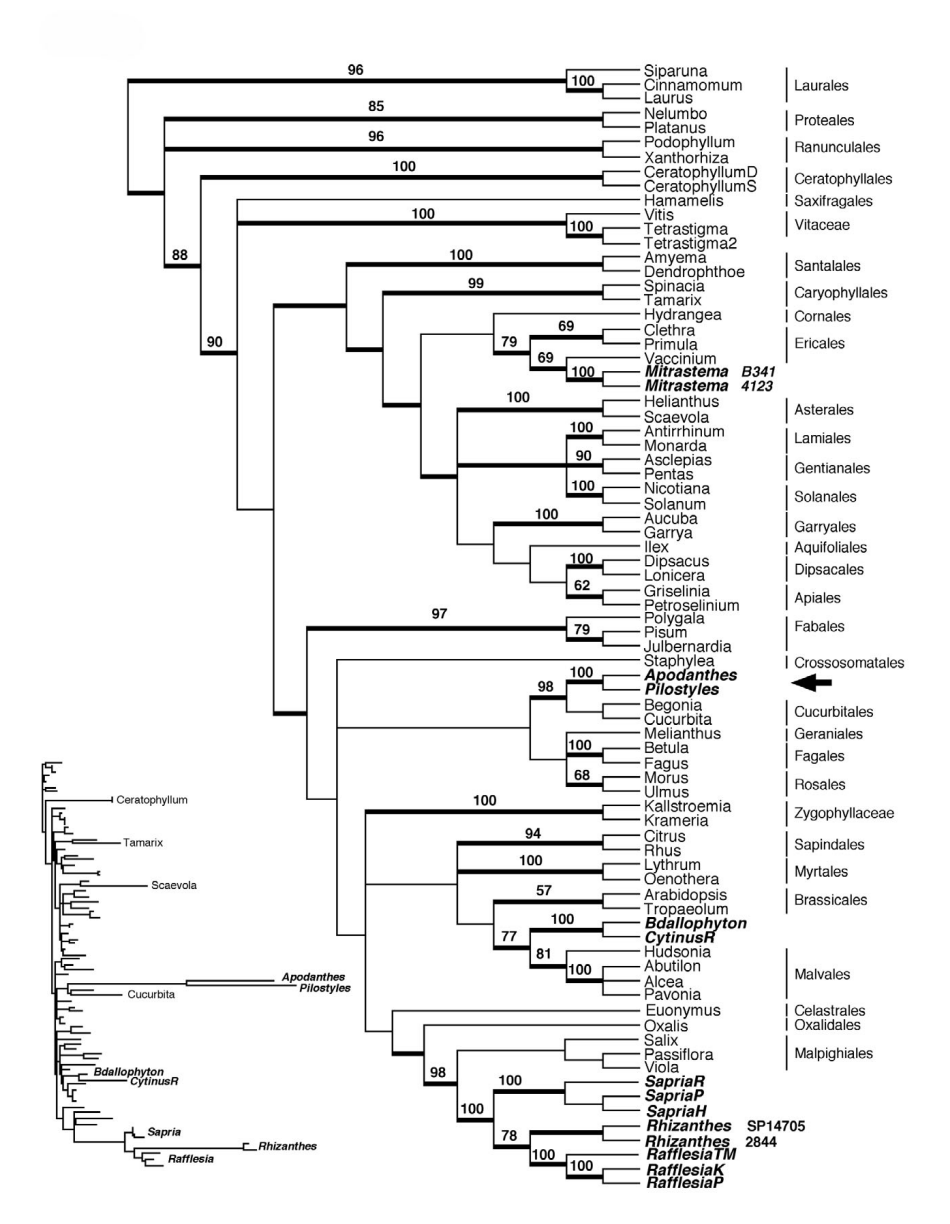
**ML strict consensus tree from mitochondrial *matR*. **Strict consensus of two trees obtained from ML analysis of the 77-taxon mitochondrial *matR *matrix. Clades with Bayesian posterior probabilities between 0.9 and 1.0 are indicated by thick lines. Bootstrap percentages from MP analysis shown above lines. Rafflesiales taxa are shown in bold italics. Arrow represents a putative cases of horizontal gene transfer. The small phylogram is included to demonstrate branch length heterogeneity.

Maximum likelihood (ML), maximum parsimony (MP) and Bayesian inference (BI) analyses of mitochondrial *matR *resulted in trees congruent with each other and with those previously generated [9] (Figure 1 and additional data file 1). As shown on the ML tree (Figure 1), *Rafflesia*, *Rhizanthes*, and *Sapria *were placed with strong support in Malpighiales. *Mitrastema *was placed in Ericales sister to *Vaccinium*. The *Cytinus *and *Bdallophyton *clade (Cytinaceae) was strongly supported and this clade was sister to one composed of four genera of Malvales, an order that contains cotton (*Gossypium*), rockrose (*Cistus*) and chocolate (*Theobroma*). For Apodanthaceae, *Apodanthes *and *Pilostyles *were sister taxa and derived from within Cucurbitales, an order that contains squash/pumpkin (*Cucurbita*) and *Begonia*. For *Berlinianche*, sequences homologous to *matR *could not be obtained using several primer combinations.

**Figure 2:**
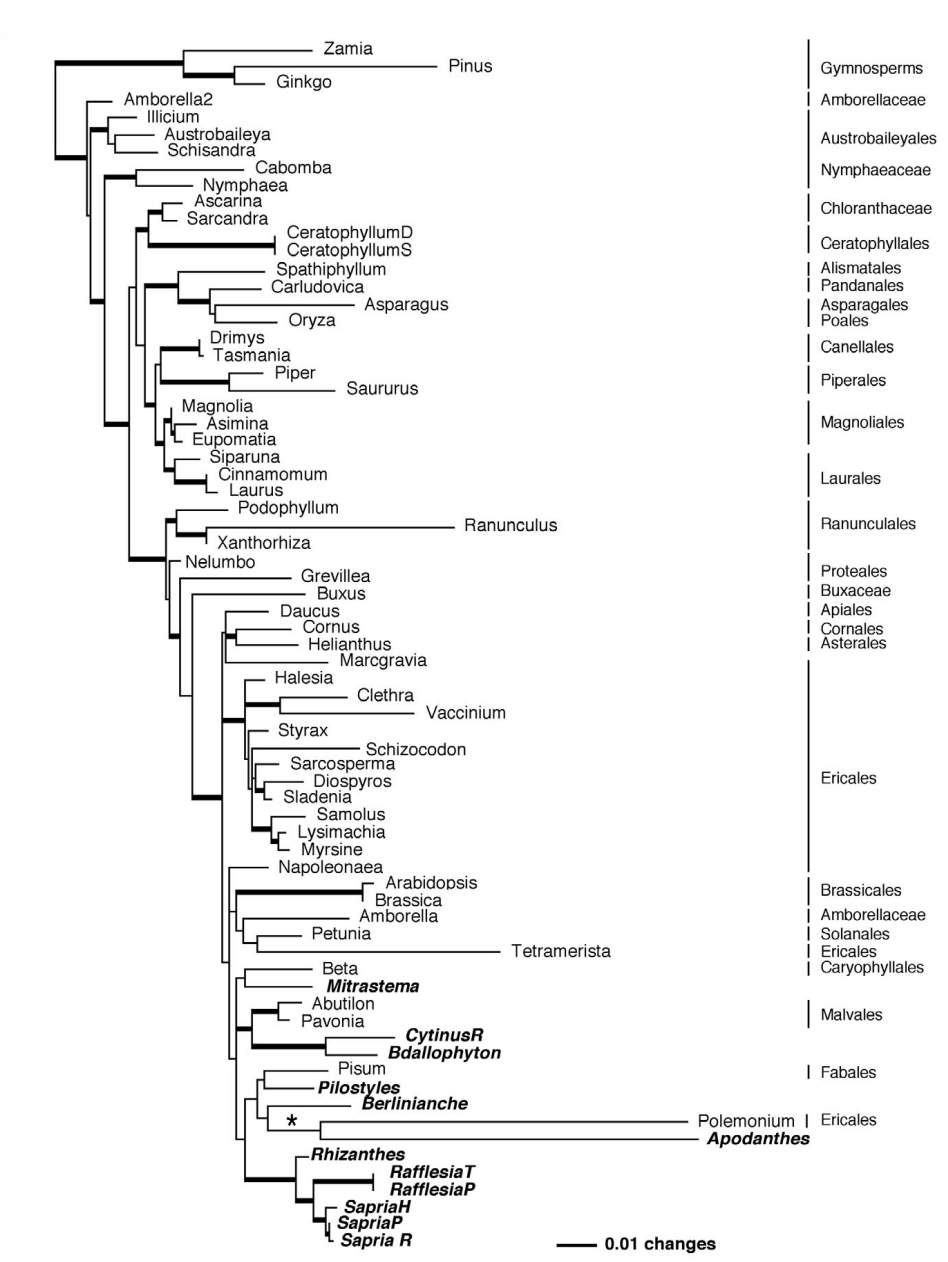
**ML tree from mitochondrial *atp1*. **Phylogram obtained from ML analysis of the 71-taxon mitochondrial *atp1 *matrix. Clades with Bayesian posterior probabilities between 0.9 and 1.0 are indicated by thick lines. Rafflesiales taxa are shown in bold italics. Note that the clade with *Apodanthes *and *Polemonium *(asterisk) is poorly supported with a posterior probability of 0.54.

All three analytical methods of the *atp1 *data produced trees that were generally congruent, thus the ML tree is illustrative (Figure 2, additional data file 2). Clades among the monosulcates generally follow previously reported relationships, whereas the topology of the eudicot portion of the tree does not clearly reflect accepted clades, possibly owing to poor sampling within rosids and asterids (sequences for these taxa were not available from GenBank). Despite these shortcomings, this gene provides additional evidence useful in assessing the phylogeny and molecular evolution of Rafflesiales. With all three analytical methods, *Mitrastema *forms a clade with *Beta *(Caryophyllales), although this relationship does not receive strong support. This is remarkable given that 15 taxa from Ericales were included, yet a relationship with this order (as seen with *matR*) was not obtained with *atp1*. The large-flowered clade was strongly supported as monophyletic in all analyses, however, its position within the eudicots did not receive strong support. Parsimony analysis placed *Pilostyles *as sister to *Pisum *(Fabales) and this clade was sister to *Berlinianche*, but both with low bootstrap support. *Apodanthes *was strongly suported (90% bootstrap) as sister to *Polemonium *(Ericales) with MP but with ML this long-branch clade received lower support (Figure 2). The two genera of Cytinaceae, *Cytinus *and *Bdallophyton*, were sister to Malvales, with moderate (MP) to strong (BI) support.

**Figure 3:**
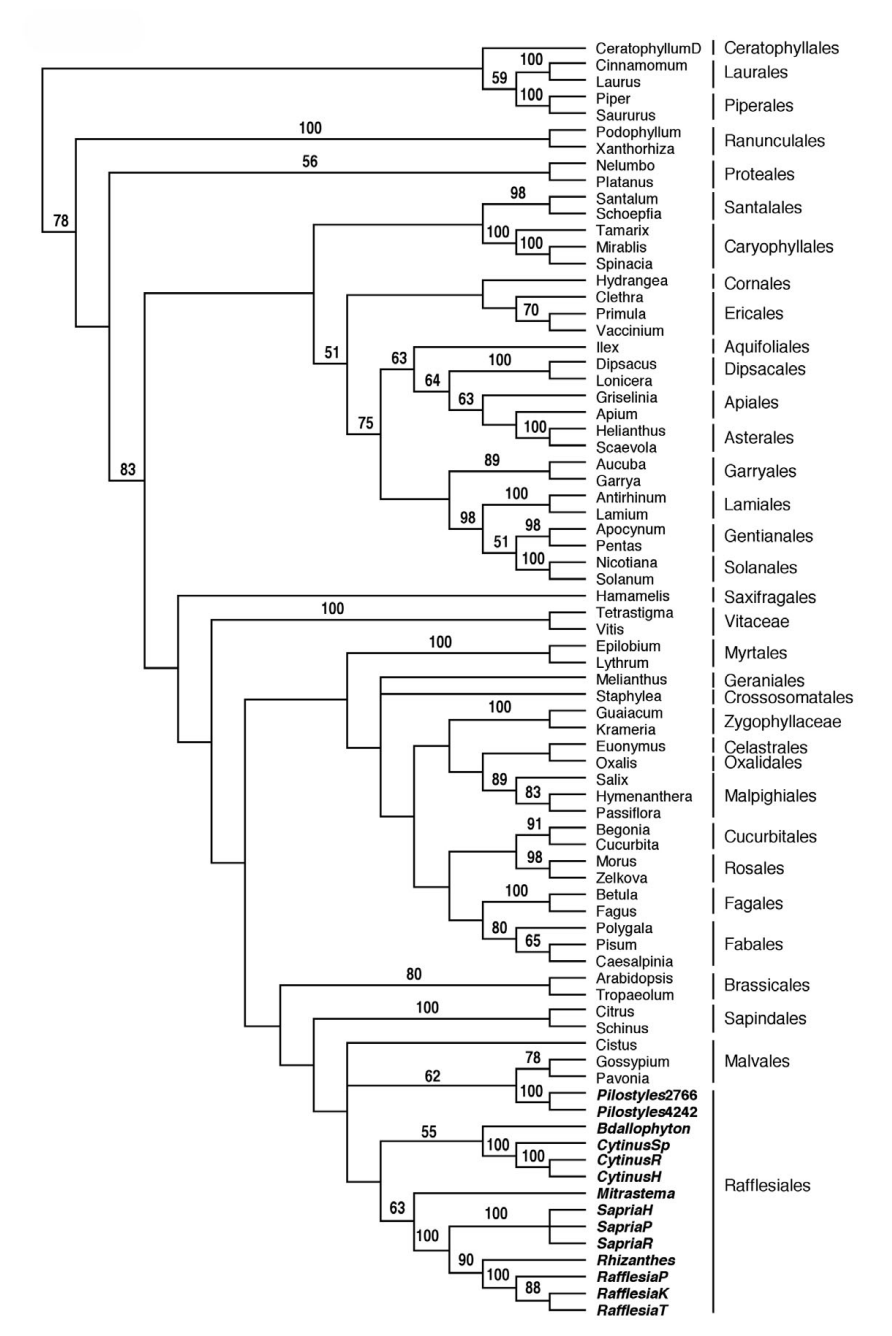
**Unconstrained MP tree from the 3-gene data matrix. **Strict consensus of 12 trees obtained from an unconstrained maximum parsimony analysis of the 77-taxon "3-gene" matrix (nuclear SSU rDNA, *rbcL*, *atpB*). Bootstrap support is shown above the lines. Rafflesiales taxa are shown in bold italics.

Maximum parsimony analyses of the full-length (103 taxon) and reduced (77 taxon) 3-gene matrices were generally congruent and both resulted in all taxa of Rafflesiales being associated with Malvales (Figure 3), although with low bootstrap support for the monophyly of this clade. The two accessions of *Pilostyles *were sister to a clade composed of *Pavonia *and *Gossypium*, also with low bootstrap support. In constrast, BI analysis of the 3-gene matrix placed *Mitrastema *with Ericales and the large-flowered clade was a component of Malpighiales, the latter with strong support. The inflorescence clade (*Cytinus *and *Bdallophyton*) and the small-flowered clade (*Pilostyles*) were allied with Malvales (see additional data file 3), although posterior probablilities of this association were lower.

**Figure 4:**
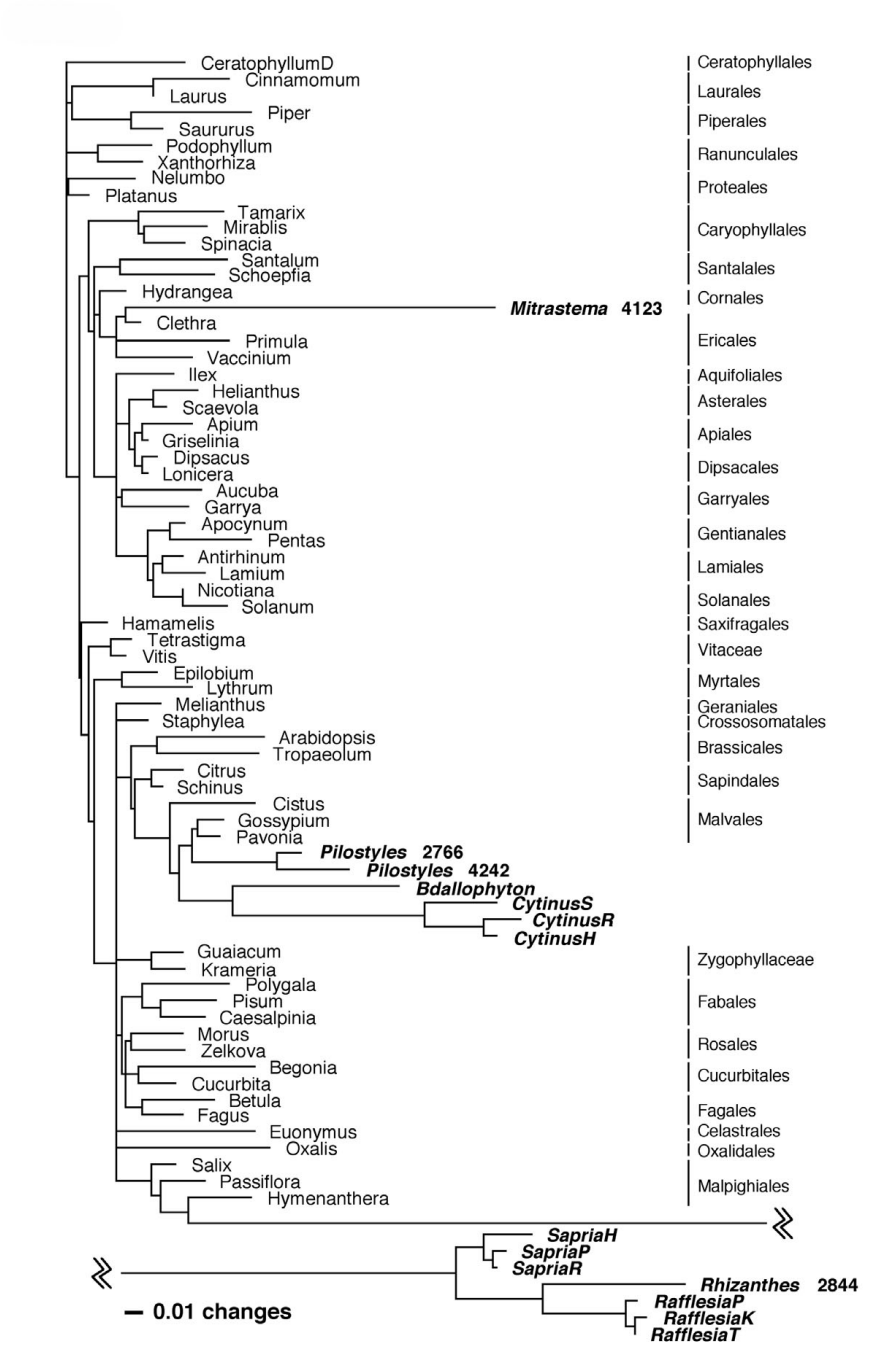
**Constrained ML tree from nuclear SSU rDNA. **Tree resulting from the constrained ML analysis of the 77-taxon nuclear SSU rDNA matrix. Rafflesiales taxa are shown in bold italics.

Parsimony analysis of the nuclear SSU rDNA matrix, constrained to an accepted topology for nonparasites, showed the same pattern of relationships as the unconstrained 3-gene MP analysis, i.e., all Rafflesiales taxa were associated with Malvales (see additional data file 4). In contrast, the tree (Figure 4) resulting from ML analysis using the same constraint tree showed the same relationships as the BI tree for the 3-gene data set.

**Figure 5:**
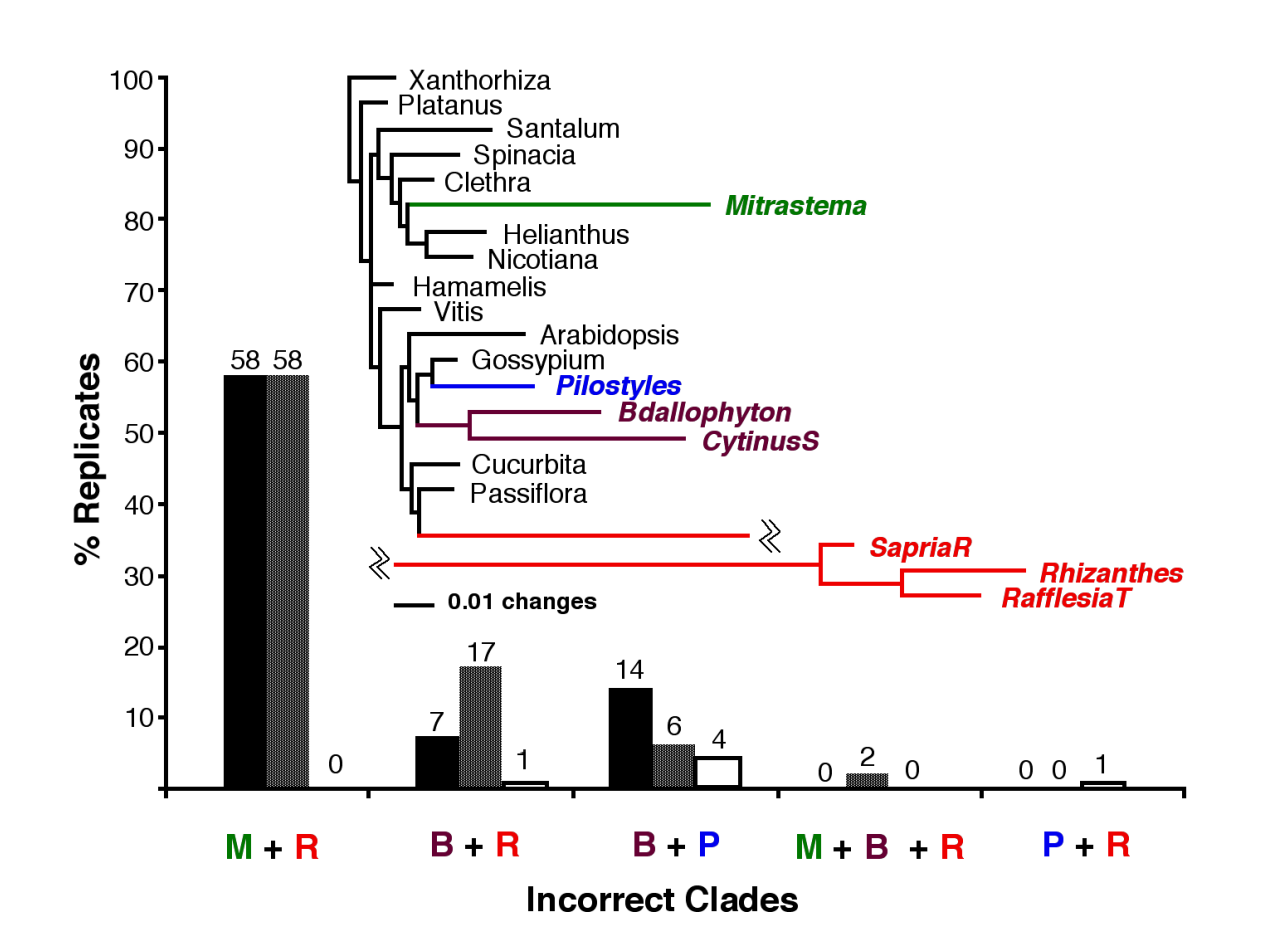
**Rafflesiales long branches mislead MP. **Proportion of simulated data sets (replicates) for which incorrect "long-branch" clades are recovered in maximum parsimony (black bars, 77 taxa), maximum parsimony (grey bars, 20 taxa), and maximum likelihood (open bars, 20 taxa) analyses. Inset is the model tree used to generate the simulated data sets. M = *Mitrastema*, B = *Bdallophyton *+ *Cytinus*, R = *Rafflesia *+ *Rhizanthes *+ *Sapria*, P = *Pilostyles*.

None of the consensus trees generated from MP analysis of the 100 nuclear SSU rDNA data sets simulated on 20-taxon trees matched the topology of the model tree. 58 of the 100 MP consensus trees showed a *Mitrastema *+ *Rafflesia/Rhizanthes/Sapria *clade and 17 showed a *Bdallophyton/Cytinus *+ *Rafflesia/Rhizanthes/Sapria *clade (Figure 5). Two other combinations, *Bdallophyton/Cytinus *+ *Pilostyles *and *Bdallophyton/Cytinus *+ *Mitrastema *+ *Rafflesia/Rhizanthes/Sapria *accounted for 6% and 2% of the MP consensus trees, respectively. Thus, 83% of the MP trees contained incorrect clades, and most of these can be attributed to the long-branch *Rafflesia *clade. However, only two of the 100 MP trees showed all six long-branch taxa as monophyletic, a result seen on the original MP tree for the full 77-taxon data set. Results of parsimony analyses of data sets simulated on the full 77-taxon tree showed a similar pattern – 58 of the MP consensus trees showed a *Mitrastema *+ *Rafflesia/Rhizanthes/Sapria *clade, 7 showed a *Bdallophyton/Cytinus *+ *Rafflesia/Rhizanthes/Sapria *clade, and 14 showed a *Bdallophyton/Cytinus *+ *Pilostyles *clade (Figure 5). In other words, MP returned an incorrect "long-branch" clade for 79% of the data sets simulated on the full 77-taxon model tree. In contrast, far fewer incorrect long-branch clades were recovered by ML for the 20-taxon simulations, and most (56%) ML trees matched the model tree in that the *Rafflesia *clade was sister to *Passiflora*, *Mitrastema *was sister to *Helianthus/Nicotiana*, and *Pilostyles*, *Bdallophyton *and *Cytinus *were associated with *Gossypium*.

MP analyses of SSU data sets from which all but one parasite group had been removed resulted in phylogenetic placements that matched those found in the ML tree. MP analysis of a data set from which all Rafflesiales except *Mitrastema *had been removed resulted in trees that placed *Mitrastema *in Ericales. Removal of all parasites except *Pilostyles *or *Bdallophyton *+ *Cytinus *individually placed both of these groups in Malvales. Finally, removal of all parasites except the large-flowered clade (*Rafflesia*, *Rhizanthes *and *Sapria*) placed this clade in Malpighiales. Thus, the positions of the parasite clades inferred in four separate MP analyses matched the positions found for these clades in the single ML tree.

## Discussion

### Rate heterogeneity and long-branch attraction artifacts

Determining the photosynthetic relatives of Rafflesiales has long presented a challenge owing to the extreme reduction and/or modification of morphological structures that have accompanied the evolution of this lineage [3,11]. Molecular phylogenetic approaches, although providing great promise in resolving such questions, also come with their own set of challenges that includes losses of some genes, substitution rate increases in other genes, and horizontal gene transfer. Examples of the first process can be seen in chloroplast genes such as *rbcL *that are typically used to infer phylogenetic relationships among angiosperms but have not yet been amplified from any Rafflesiales and are presumed lost [5]. Increased substitution rates in the normally conservative plastid rDNA has been demonstrated in these holoparasites [4,12]. Similarly, accelerated rates in mitochondrial SSU rDNA, typically very conservative in many photosynthetic angiosperms, occur in *Rafflesia *and *Cytinus *[13]. Despite these complications, molecular phylogenetic analyses of some holoparasite lineages with comparatively lower rates have been tractable. For example, the mitochondrial genes *atp1 *and *matR *were used, in combination with nuclear rDNA and chloroplast genes, to reliably place Hydnoraceae with Aristolochiaceae [11].

Long-branch attraction, a bias in certain phylogenetic inference methods in which similarity due to convergent or parallel changes produces an erroneous phylogenetic grouping of taxa [10], is often implicated as the reason for anomalous phylogenetic groupings [14]. It has been suggested that some data sets with marked among-lineage rate heterogeneity cannot be applied to particular phylogenetic problems owing to hypothesized long-branch attraction artifacts [15]. In their unconstrained parsimony analysis of several angiosperm SSU rDNA sequences, Barkman et al. [9] found that the branch leading to *Rafflesia *was several times longer than any other branch, and that this branch was attracted to the second-longest branch in the tree – the one between gymnosperms and angiosperms. For these reasons, they argued that nuclear SSU rDNA sequences are of limited utility for assessing the phylogenetic position of *Rafflesia*.

Barkman et al. [9] analyzed their SSU rDNA data using only parsimony, not model-based methods (e.g., ML or BI methods) that are less likely to be misled by long-branch attraction [16]. Our ML analysis of the SSU rDNA data recovers a topology that closely matches the *matR *topology presented by Barkman et al. [9] in which *Rafflesia *is closely related to Malpighiales and *Mitrastema *is a member of Ericales (Figure 4). These results highlight the requirement to analyze SSU rDNA data with methods less biased by long-branch attraction than parsimony, as well as the advantage gained by independent confirmation of results obtained from a single gene.

Several authors have suggested that adding taxa can "break up" long branches and allow parsimony to recover the correct topology [17-19]. Our parsimony analysis of the 103- and 77-taxon SSU rDNA data sets, in which we included representatives of all genera of Rafflesiales (i.e., sequences that could potentially break the *Rafflesia *long branch), recovers a nearly monophyletic Rafflesiales containing all of the longest terminal branches in the tree (see additional data file 3). Based on our simulation study and MP analyses of data sets from which all but one parasite group was removed, we believe that this topology represents a case of long-branch attraction. These simulation results support the contention that the branches leading to the parasitic taxa are long enough to attract one another (Figure 5), a result in agreement with previous work [3,6].

Taxon sampling is not a cure-all for long-branch attraction problems [20]. Even for the data sets simulated on the full 77-taxon tree, MP returned incorrect long-branch clades nearly 80% of the time. MP did nearly as poorly with data sets simulated on a 77-taxon tree as it did on data sets simulated on a 20-taxon tree. Evaluation of the ML tree for the SSU data (Figure 4) shows that increasing the number of taxa from 20 to 77 did not improve the result because the long parasite branches were not broken. Instead, shorter (nonparasite) branches were broken which did not help MP recover the true topology for the simulated data sets. MP analyses of the full 77-taxon SSU data set that included all parasite clades resulted in a worse estimate of the phylogeny than MP analyses of smaller data sets in which only single parasite clades were included. Thus, the frequently stated view that increased taxon sampling can help MP avoid long-branch attraction problems may only be true if the added taxa are not distantly related long-branch clades themselves.

### Phylogenetic relationships of the four Rafflesiales clades

#### Rafflesiaceae (the large-flowered clade)

The results from analyses of Rafflesiales using independent data sets are summarized in Table 1. For Rafflesiaceae s. str., placement in Malpighiales is supported by ML and BI analyses of the 3-gene and nuclear SSU rDNA data sets as well as mitochondrial *matR*. This placement in Malpighiales is also supported by a molecular phylogenetic study that used a single copy nuclear gene *phytochrome C *[21]. These authors proposed that Rafflesiaceae are most closely related to Ochnaceae or Clusiaceae which contrasts with presumed synapomorphies with *Passiflora *given by Barkman et al. [9]. Within Malpighiales, tremendous morphological diversity exists among the 27 families and 16,000 species. Moreover, relationships among the major clades are still poorly resolved [22]. Although the evidence for a malpighialean affinity of Rafflesiaceae appears strong, it is possible that the molecular data have only identified the stem group that represents the sister to the parasitic lineage.

**Table 1:** Summary of phylogenetic analyses of Rafflesiales using different data partitions and methods of analysis.

	3-Gene*	**3-Gene**	**nuSSU rDNA**	**nuSSU rDNA**	***matR***	***matR***	***atp1***	***atp1***
	**Parsimony**	**Bayesian**	**Parsimony constrained**	**Likelihood constrained**	**Parsimony**	**Likelihood & Bayesian**	**Parsimony**	**Likelihood & Bayesian**

*Mitrastema*	*Malvales*	Ericales	*Malvales*	Ericales	Ericales	Ericales	Caryophyllales	Caryophyllales
*Cytinus*	Malvales	Malvales	Malvales	Malvales	Malvales	Malvales	Malvales	Malvales
*Bdallophyton*	Malvales	Malvales	Malvales	Malvales	Malvales	Malvales	Malvales	Malvales
*Apodanthes*	N/A	N/A	N/A	N/A	Cucurbitales	Cucurbitales	*Polemonium*	*Polemonium*
*Pilostyles*	Malvales	Malvales	Malvales	Malvales	Cucurbitales	Cucurbitales	Fabales	Fabales
*Berlinianche*	N/A	N/A	N/A	N/A	N/A	N/A	Ericales/Fabales	Ericales/Fabales
*Rafflesia*	*Malvales*	Malpighiales	*Malvales*	Malpighiales	Malpighiales	Malpighiales	Eudicots	Eudicots
*Rhizanthes*	*Malvales*	Malpighiales	*Malvales*	Malpighiales	Malpighiales	Malpighiales	Eudicots	Eudicots
*Sapria*	*Malvales*	Malpighiales	*Malvales*	Malpighiales	Malpighiales	Malpighiales	Eudicots	Eudicots

*Nuclear SSU rDNA plus chloroplast *rbcL *&*atpB*.

Possible HGT events

Long-branch artifact

Barkman et al. [9] suggested that the floral similarities between *Rafflesia *and *Passiflora*, first noted by Robert Brown [23] represent morphological synapomorphies that support the results obtained from the *matR *gene tree. Arguments in favor of a number of other, equally credible relationships within eudicots could be made based on hypothetical evolutionary transformation series of morphological characters. Indeed Brown concluded that *Rafflesia *may have affinity with Passifloraceae (Malpighiales) but he also considered other groups such as Aristolochiaceae ("Asarinae", Piperales), Sterculiaceae (Malvales) and Cucurbitaceae (Cucurbitales). In general, different characters supported relationships with one or another group and therefore he left the subject as unresolved. Three proposed synapomorphies between Passifloraceae and *Rafflesia *were cited by Barkman et al. [9]: a hypanthium (perigone tube in *Rafflesia*), an androgynophore (gynostemium or column in *Rafflesia*), and an annular corona (diaphragm in *Rafflesia*). Whether these structures are homologous is not clear and will likely require further morphological studies, possibly examining the floral development genes themselves. These hypotheses require scrutiny because the apparent similarities in structure are not clear when examined in detail. For example, the androgynophore of *Passiflora *is composed of a stalk that bears the androecium and gynoecium. In *Rafflesia*, the ovary is inferior (with no stalk), hence the central column must involve other gynoecial parts. The corona of *Passiflora *is very different in structure and function from the diaphragm of *Rafflesia *[24]. The observation of a physical union between *Passiflora caerulea *and *Euonymus *[25] was discussed by Barkman et al. [9] as a possible clue to the origin of parasitism in *Rafflesia*. Whether this association represents parasitism or not is a matter of semantics [26], for other similar associations exist such as *Cissus *and *Opuntia *growing on *Yucca *and *Opuntia *on *Cercidium *and *Idria*. In all of these cases, a true haustorium does not form and more likely these represent forms of grafting. It is difficult to state whether such rare occurrences have any bearing on the origin of parasitism in Rafflesiales or other parasitic flowering plants.

#### Mitrastemonaceae (the hypogynous clade)

Maximum likelihood and Bayesian analyses of the 3-gene and nuclear SSU rDNA data partitions placed *Mitrastema *in Ericales, a result congruent with that obtained using mitochondrial *matR*. As noted by Barkman et al. [9], this relationship within the asterids had not previously been proposed. *Mitrastema *has bisexual, protandrous flowers with a collar-shaped, four-merous perianth tube. The stamens are connate into a tube (androphore) crowned by a fertile zone of pollen-bearing locules. The staminal tube, open at the top by a small hole, circumscissally separates from the flower as it is pushed up by the growing gynoecium. The apical portion of the staminal tube is sterile, but below this is a series of vertical rings of ca. ten minute, pollen sacs each. The gynoecium is hypogynous, one-locular, with a thick, conical stigma. Placentation is parietal with 8–15 (-20) unequal placental lobes filling the locule. The numerous ovules are small (190 by 120 μm), anatropous, unitegmic (but with two cell layers), and tenuinucellar. Although some floral morphological features of *Mitrastema *are not in conflict with those seen in Ericales, such as extrorse anthers and cellular endosperm, features such as decussate leaves, circumscissile fruit dehiscence, and parietal placentation are too general to draw specific associations. Given that *Mitrastema *is an achlorophyllous holoparasite and that one clade of Ericaceae (Monotropoideae) contains achlorophyllous mycotrophs, it is intriguing to ask whether these groups share a common ancestor or evolved independently. The most specialized morphological feature found in Mitrastemonaceae, the athecal androecium, is not found in Ericales but in Malvaceae, the only angiosperm family that shows the entire gamut from taxa with normal stamens, to taxa with stamens deviating only slightly from the common pattern [27,28], to athecal androecia [29].

#### Cytinaceae (the inflorescence clade)

The most consistent phylogenetic signal that is seen across all data sets and types of analyses is a relationship between Cytinaceae and Malvales (Table 1). Because the relationship between Cytinaceae and Malvales is the strongest among all four Rafflesiales clades, it is possible that this clade acts as an "attracter" for the other three Rafflesiales clades in some analyses. This is seen when using nuclear SSU rDNA sequences, either alone or with the topology of the tree stabilized through the addition of two chloroplast genes. In both cases, parsimony produces a monophyletic Rafflesiales within Malvales which contrasts with the result seen with the constrained ML SSU rDNA and the *matR *results. These results and those obtained from the simulation study indicate that the large-flowered clade and *Mitrastema *are artifactually attracted to Cytinaceae when parsimony is utilized.

Unlike other Rafflesiales, members of Cytinaceae have multiple flowers arranged in an inflorescence. The floral structure called the diaphragm, seen in *Rafflesia *and *Sapria *(but not *Rhizanthes*), is lacking in Cytinaceae. *Bdallophyton *is dioecious and *Cytinus *is either dioecious (*C. capensis*, *C. sanguineus*) or monoecious (*C. hypocistis*). The perianth is tubular, composed of four to nine imbricate organs. The androecium is connate, forming a compact synandrium with extrorse anthers and the pollen is 2-, 3-, or 4-porate. The female flower is epigynous with a columnar style terminated by a globose or capitate, viscous stigma with commissural lobes [30]. The ovary is unilocular with 8–14 deeply intrusive, discrete parietal placentae that bear numerous, orthotropous, tenuinucellate ovules.

#### Apodanthaceae (the small-flowered clade)

Maximum parsimony and likelihood analyses of the 3-gene data set and nuclear SSU rDNA sequences alone also place *Pilostyles *(the only Apodanthaceae for which SSU rDNA sequences are available) within Malvales, however, a sister relationship with Cytinaceae is not consistently obtained. A 3-gene alignment that included additional representatives of Malvales (16 taxa) gave similar results as shown in Figure 3 (i.e., *Pilostyles *on a clade separate from other Rafflesiales). These data, in conjunction with the results from the mitochondrial genes, support an evolution of Apodanthaceae independent from Rafflesiaceae s. str. The well-supported relationship between *Pilostyles *and *Apodanthes *using *matR *is expected given their very similar floral morphology [31], yet this clade is sister to two representatives of Cucurbitales (*Begonia *and *Cucurbita*). Contamination with host tissue is excluded because neither parasite is known to currently occur on a member of Cucurbitales. Apodanthaceae are grouped with *Pisum *(Fabales) and *Polemonium *(Ericales) on the *atp1 *tree, but no *atp1 *sequences from representatives of Cucurbitales were available from GenBank to test the *matR *result. The sister relationship between *Apodanthes *and *Polemonium *is strongly supported on the MP tree (bootstrap support value = 90%; additional data file 3), but this pairing must be viewed with caution given the low Bayesian posterior probability of the clade (0.54) and that both taxa are very long branches (Figure 2). Although ML is less susceptible to long-branch attraction artifacts than MP, it is not immune to it; thus, it remains unclear whether or not this relationship is artifactual. Moreover, the *Polemonium *sequence is separate from the clade containing 12 other members of this order, thus raising the possibility that the sequence results from contamination or HGT (see below). Additional sampling within the eudicots will be required to better understand the *atp1 *gene tree topology.

Morphological features shared between Apodanthaceae and Cytinaceae are: unisexual flowers, a connate androecium, an inferior ovary, and a unilocular ovary with four parietal placentae bearing numerous, anatropous, tenuinucellate ovules [30,31]. Floral morphological features that might link Apodanthaceae and Cytinaceae with Malvales [31] include an androecial tube (e.g., Malvaceae), a trend toward synandria without anthers and thecae (e.g., Malvaceae) [29], tri- to hexamerous flowers (e.g., Thymelaeaceae), and parietal placentae (e.g., Cistaceae). The floral conditions of unisexuality and epigyny do occur in Malvales, albeit rarely. Unisexual flowers pose some difficulties for interpreting the morphological homologies of various floral organs. For *Pilostyles *and *Apodanthes *male flowers, a tubular synandrium surrounds and fuses with a central structure that could be interpreted as a sterile gynoecium. Support for the concept that such a central structure is a pistillode comes from *Berlinianche *where the upper portion of the synandrium is free from the central part. In female flowers of Apodanthaceae, there is no rudiment of an androecium, hence the central tissue is apparently entirely gynoecial.

In contrast to the above discussion, the *matR *data indicate Apodanthaceae are related to Cucurbitales, an order with seven families, 129 genera and 2300 species. Hosts for Apodanthaceae are generally legumes, although *Apodanthes *occurs most frequently on *Casearia *(Salicaceae, Malpighiales). Thus, neither recent HGT nor contamination explains this result. Apodanthaceae shares some morphological features with members of Cucurbitaceae, subfamily Cucurbitoideae: unisexual, five-merous flowers (*Berlinianche*); carpellate flower with a unilocular, inferior ovary with parietal placentation; anatropous, bitegmic ovules; staminate flower with connate filaments (monadelphous) and a rudimentary gynoecium (pistillode) [32]. Conflicting characters also occur, such as a three-carpellate gynoecium in Cucurbitoideae (vs. four-carpellate in Apodanthaceae) and a valvate perianth (vs. imbricate). All of these characters, however, are less specialized than those shared between Apodanthaceae and Malvales.

### Background on horizontal gene transfer

A requirement of the molecular phylogenetic approach to inferring evolutionary histories of organisms is vertical transmission of genetic material from parent to offspring. In contrast, horizontal gene transfer (HGT) describes the movement of genetic material between organisms of no direct ancestor-descendant relationship. Although the frequency of HGT is currently not well understood among prokaryotic and eukaryotic organisms, it is clear that HGT can compromise accurate inference of genealogical history. In plants, lateral movement of genetic material has been documented for mobile genetic elements such as introns [33-37] but only recently has convincing evidence emerged documenting HGT of mitochondrial genes [38,39]. Genes of the mitochondrion are extensively used to infer evolutionary relationships in plants [40-42], thus highlighting the importance of characterizing the frequency of HGT across genes and taxa.

Incongruence among gene trees derived from different data sets can derive from a number of factors such as technical causes (insufficient data, gene choice, sequencing error, taxon sampling and identification), gene/genome-level processes, and organism-level processes (e.g., hybridization/introgression, lineage sorting, and HGT) [43]. HGT has only recently been recognized as a potentially important force in the evolution of plant mitochondrial genomes and detecting HGT is highly dependent upon the presence of multiple gene data sets with robust taxon sampling [38,39].

### Evidence for horizontal gene transfer in parasitic plants

We believe that incongruence between the the mitochondrial and the nuclear gene trees (Table 1) stem not just from long-branch attraction artifacts but also from cases of HGT. The placement of *Apodanthes *and *Pilostyles *on the *atp1 *tree as sister to *Pisum *(a legume, the family of hosts for *Pilostyles*) represents a likely case of HGT. The *atp1 *data conflict with those from *matR *that associates Apodanthaceae with Cucurbitales. Moreover, we infer that the SSU rDNA tree better represents the organismal phylogeny because it seems less likely that nuclear genes would be influenced by HGT [44,45]. The main rationale for this is that nuclear rDNA cistrons are repeated hundreds to thousands of times in tandem arrays at nucleolar organizing regions of the chromosomes. Although it can be envisioned that concerted evolution could homogenize all rDNAs in the parasite with a form obtained via HGT, the probability of this happening is small given the vastly different number of starting copies.

In their study of Rafflesiaceae s. str. and Mitrastemonaceae, Barkman et al. [9] discounted HGT as a possible explanation for their results because they state the phenomenon is rare and the overall topology of the *matR *tree closely matched results from other molecular phylogenetic investigations of angiosperms. The present study confirms that HGT is not implicated for the two lineages studied by Barkman as well as Cytinaceae, but this process could be invoked for Apodanthaceae. More recent work by these authors [46] identified several cases of HGT from host to parasite for *atp1*. These included *Dalea *to *Pilostyles*, *Tetrastigma *to *Rafflesia*, and *Lithocarpus *to *Mitrastema*. In addition, HGT of another mitochondrial gene, *nad1*, has been reported for *Rafflesia *and *Sapria*, both of which occur on the same clade as their hosts (*Tetrastigma*) on a gene tree [20]. These examples demonstrating the presence of host genes in parasitic plants provide the most compelling evidence for HGT. This form of transfer is intuitively logical given the intimate contact between cells of the two organisms via the endophytic haustorium. However, parasitic plants exist in complex ecosystems where they are in physical contact with many other organisms (bacteria, fungi, phytophagous and pollinating animals, etc.) that could potentially affect HGT. That such nonhost HGT may also be occurring is evidenced by the presence of an apparent cucurbitalean *matR *gene in *Pilostyles *and *Apodanthes*. Moreover, present-day hosts of parasitic angiosperms do not represent the only conduit for HGT if host choice has shifted through time as the parasite lineage evolves. For example, Barkman et al. [9] state that *Mitrastema *only parasitizes Fagales (e.g., *Lithocarpus *and *Castanopsis*, both Fagaceae) but this parasite has also been recorded from Aquifoliaceae, Asteraceae, Elaeocarpaceae, Juglandaceae, and Myrtaceae [47]. Host latitude for this species would be broader if rare hosts and hosts of parasite ancestors were fully known, thus expanding the taxonomic spectrum of potential HGT sources.

### Formidable contamination issues

Contamination of parasite DNA with DNA from the host plant is an issue that must be given serious attention. Indeed, two sequences shown on the *matR *tree (Figure 1), *Tetrastigma2 *and *Julbernardia *are hosts for *Rafflesia tuan-mudae *and *Berlinianche*, respectively. These sequences were obtained by PCR amplification and sequencing from what was originally thought to be pure parasite genomic DNA. Sequences of the host (obtained from separate samples) were found to be identical to these "parasite" sequences, strongly suggesting contamination. In the case of *Rafflesia*, the DNA was obtained from a bud still attached to the host vine, both of which had been sectioned longitudinally. Disruption of these tissues likely resulted in transfer of host sap to the bud region where the tissue was sampled. Other samples of *R. tuan-mudae *from the same population, obtained as floral bracts with no host tissue, resulted in *matR *sequences that were similar to the other two *Rafflesia *species.

For *Berlinianche*, whose flowers are much smaller than those of *Rafflesia *(5 mm in diameter), extreme care (using a stereo microscope) was exercised to remove floral parts devoid of any host tissue. Despite this, the *matR *sequence obtained from the first sample was that of the host, *Julbernardia*. Later, silica gel dried samples of other populations of the parasite were extracted, again using extreme care in avoiding host contamination. PCR products were obtained using several mitochondrial *matR *primers, but none were found to be homologous to this gene following BLAST searches. This result shows that host DNA was not present in this sample in sufficient amounts to amplify and that the parasite *matR *gene, if present, is highly divergent at the priming sites used.

For all three Apodanthaceae genera, the conical style in female flowers is papillate and heavily secretory [31]. This sticky surface tends to capture a variety of environmental debris, likely including extraneous pollen, fungal spores, and host tissues that have been disrupted upon collecting. Obtaining a proper nuclear SSU rDNA sequence for *Pilostyles *was extremely difficult. Despite PCR products of the correct sizes using a variety of primer combinations, the sequences obtained from genomic DNA derived from flowers were deemed contaminants following BLAST searches that showed them to be most similar to monocots, fungi, etc. Only when sequences from two accessions of *Pilostyles *(Texas and California) both were most similar to Malvales was this considered good evidence for their true phylogenetic affiliation. Retrospectively, it is likely that the sticky flowers had accumulated wind-dispersed pollen (e.g., grasses) and that this DNA, despite being in low concentration, had less divergent priming sites than the parasite target DNA, allowing PCR to preferentially amplify the contaminant DNA.

### The mechanism of horizontal gene transfer: some considerations

Given the accumulating molecular evidence for HGT from host to parasitic plant, it is worthwhile to consider potential mechanisms, along with their constraints, that may suggest further research. Relatively little information exists on the structure of the endophyte of Rafflesiales. Ultrastructural studies have been conducted on two species of *Pilostyles*: *P. hamiltonii *[48] and *P. thurberi *[49]. These authors conflict, however, as to whether there exists symplastic continuity between host and parasite via plasmodesmata; the former indicated that such connections are the major path of nutrient uptake by the parasite whereas the latter rejected this idea. Despite this controversy, heteroplastic plasmodesmatal connections have been documented in another parasitic plant, *Cuscuta *[50] and indeed such connections can even form in heterografts between distantly related plant taxa [51]. Given this, we assume that host-parasite plasmodesmatal connections exist in the endophytes of Rafflesiales. Transmission electron micrographs of *Pilostyles *suggest that intact, mature mitochondria are too large to pass through heteroplastic plasmodesmata, however, mitochondrial genomes or portions of the genome are certainly small enough for transmission. Once inside the parasite cell, there are various fates for the host gene. It could become incorporated into the parasite mitochondrial genome, and then either replace the parasite copy or exist as a duplicate, or the host gene could reside in the parasite nuclear genome. For the latter case, the gene would likely become a pseudogene given the requirement of mitochondrial-specific patterns of RNA editing. Two forms of *atp1 *are present in the primitive angiosperm *Amborella trichopoda *[38], one of which is derived from a HGT event from a eudicot. It is not known whether both forms of the gene exist in a single mitochondrial genome, in different mitochondrial genomes within the cell (i.e., heteroplasmy), or if one is nuclear and the other mitochondrial. Future work to address these questions would involve sequencing flanking regions of purported horizontally transferred genes to determine their subcellular location. Additionally, cDNA sequences obtained from *matR *mRNA would be useful to determine whether the gene is expressed and whether mitochondrial-specific RNA editing patterns are present.

## Conclusions

In this study we have used data derived from nuclear, mitochondrial and chloroplast DNA and a variety of analytical approaches to address long-standing questions about the holoparasitic flowering plant order Rafflesiales. We show that Rafflesiales are not monophyletic but composed of at least three and possibly four independent lineages. Rafflesiaceae (*Rafflesia*, *Rhizanthes*, and *Sapria*) representing the large-flowered clade are monophyletic and are related to Malpighiales. The monogeneric family Mitrastemonaceae, the only member of the order with a superior ovary, is related to Ericales. The first of the remaining two families that have previously not been sampled is Cytinaceae (*Bdallophyton *and *Cytinus*) which is strongly supported as a member of Malvales. The last remaining unsampled family, Apodanthaceae (*Apodanthes*, *Berlinianche*, and *Pilostyles*) is either related to Malvales or Cucurbitales. Our simulation studies indicate that *Mitrastema*, *Bdallophyton/Cytinus*, and *Rafflesia/Rhizanthes/Sapria *have branches that are long enough to mislead parsimony. All of these relatively long branches appear to be attracted toward the Cytinaceae clade within Malvales. When nuclear SSU rDNA sequences are analyzed with ML, results fully congruent with those previously reported for two Rafflesiales clades using mitochondrial *matR *are obtained. If the phylogenetic affinityof Apodanthaceae are with Malvales, the results from the mitochondrial *matR *gene must represent a case of horizontal gene transfer (HGT) from Cucurbitales. If this proves to be the case, this provides an example of HGT from a nonhost plant to a parasitic angiosperm.

To properly discern phylogenetic relationships in enigmatic parasitic taxa, our results demonstrate the need for robust taxon sampling, gene sequences from multiple subcellular compartments, and the use of analytical methods that accommodate rate heterogeneity and avoid the pitfalls of long-branch attraction. When the phylogenetic relationships among such holoparasitic taxa are poorly known, the strongest phylogenetic signal that can be obtained is congruence among results derived from independent sources (i.e., genes from different subcellular compartments). Comparisons among gene trees allows for the identification of HGT, a phenomenon that requires further investigation to determine its modes of action and frequency among taxa and through evolutionary time.

## Methods

### DNA extraction, PCR, sequencing

DNA was extracted, amplified, cloned, and sequenced by using methods formerly reported [52-54]. The nuclear and mitochondrial sequences were PCR-amplified using primers reported elsewhere [6,55,56] and are also given on the first author's web site [57]. Sequencing was conducted using manual and automated methods (ABI Prism^® ^377 automated DNA sequencer, Applied Biosystems) according to manufacturer's protocols.

### DNA alignments

The initial *matR *alignment incorporated all of the Rafflesiales parasites and the nonparasite sequences previously published [9] as well as our newly generated sequences. The 106-taxon matrix represented over 40 orders and contained three gymnosperm outgroup taxa (*Ginkgo*, *Pinus*, and *Zamia*), 28 monosulcates, 63 nonparasitic eudicots, and 15 Rafflesiales. For two taxa (*Mitrastema *and *Rhizanthes*), our sequences, as well as those previously published, were from the same species but different accessions to test for consistency. Taking into account codon information, an alignment of 2177 sites was constructed manually using SeAl version 2.0 [58]. The full matrix was used for parsimony analyses whereas another, truncated to 77 taxa by removing all but three monosulcate taxa (Laurales used as outgroup), was constructed to facilitate likelihood analyses. This operation was justified because monosulcates were never implicated as relatives of Rafflesiales in any analyses. A 71-taxon, 1265-site *atp1 *alignment was similarly constructed and included the same gymnosperm outgroup genera as above, 24 monosulcates, 32 nonparasitic eudicots and 12 Rafflesiales. All of the monosulcate genera in the *atp1 *alignment were also represented in the *matR *data set, whereas eudicot sampling for *atp1 *was constrained by sequences available on GenBank (12 of the same genera as with *matR *or placeholders from same family).

To test the position obtained for Rafflesiales taxa using mitochondrial genes with an independent data set derived from different compartments, a 4646-site "3-gene" matrix combining sequences from nuclear SSU rDNA and chloroplast *rbcL *and *atpB *was constructed that included 103 taxa (3 gymnosperms, 28 monosulcates, 58 nonparasitic eudicots, and 14 Rafflesiales). Sampling across angiosperm orders was very similar to the *matR *matrix, differing only by the presence of 11 placeholders and a second accession of *Pilostyles*. For the holoparasites, only nuclear SSU rDNA sequences were included; the chloroplast gene data for these taxa were coded as missing. The two chloroplast genes were included to add stability to the tree topology given that nuclear SSU has been shown to contain lower phylogenetic signal when used alone [15]. As with *matR*, the 103-taxon matrix was truncated to 77 taxa by removing all but five monosulcate taxa to facilitate likelihood analyses. All alignments reported in this paper have been deposited with TreeBASE [59]: **study accession number S1177, matrix accession numbers = M2034–M2037**.

### Data analysis

All three data sets were analyzed using maximum parsimony (MP) and maximum likelihood (ML) methods in PAUP* 4.0b10 [60] and Bayesian inference (BI) methods in MrBayes 3.0b4 [61].

#### Maximum parsimony

All MP searches were performed using 100 random addition sequence replicates with tree-bisection-reconnection (TBR) branch-swapping, holding ten trees at each addition step, with all sites equally weighted. For the 77-taxon SSU data set, a series of four MP analyses were performed in which all but one parasite group (*Bdallophyton *+ *Cytinus*, *Mitrastema*, *Pilostyles *or the large-flowered clade comprising *Rafflesia*, *Rhizanthes *and *Sapria*) was removed to determine the position of each parasite group in the absence of other long-branch parasite taxa in the analysis. This is a form of the test proposed by Siddall and Whiting [62].

#### Maximum likelihood

For ML analyses, a MP tree was used in PAUP* to evaluate 56 nucleotide substitution models. ModelTest 3.06 [63] was used to select an appropriate model from the PAUP* output using hierarchical likelihood-ratio tests (hLRT's) and the Akaike Information Criterion (AIC). The general time-reversible (GTR) substitution model with among-site rate heterogeneity modeled with a "gamma + invariant sites distribution" (Γ + I) was chosen via the AIC as the best-fitting model for the *atp1 *data set. Investigation of the likelihood score output from PAUP* suggested that a simpler model not evaluated by ModelTest was not significantly worse than the GTR+Γ + I model (LRT; p = 0.520824). This submodel employed four (rather than six) relative rate parameters: one for A-C transversions and A-G transitions, one for A-T and C-G transversions, one for C-T transitions, and one for G-T transversions; the PAUP* LSET option used for analysis was "RCLASS = (a a b b c d)". Likewise, the models chosen by ModelTest for the *matR *data set were TVM+Γ (hLRT) and TIM+Γ (AIC), but a simpler statistically equal model (LRT; p = 0.583393) was used for analysis. This model employed three relative rate parameters: one for A-C, A-G, and G-T substitutions; one for A-T and C-G substitutions; and one for C-T substitutions; "RCLASS = (a a b b c a)", with among-site rate heterogeneity modeled with a gamma distribution. These simplified models were chosen to reduce computational time and to avoid estimation of unnecessary parameters, which can lead to greater variance in parameter estimates and higher topological uncertainty.

A successive approximations approach was used for all ML analyses [19,64]. Substitution model parameters were estimated from the data on a MP tree. With parameter estimates fixed, starting trees for ML analyses were produced via random stepwise addition using five starting seeds, with each tree subjected to a round of tree bisection-reconnection (TBR) branch swapping. Substitution model parameters were then re-estimated on all resulting trees, followed by another round of random stepwise addition and TBR swapping. The tree with the highest likelihood was accepted as the ML tree.

#### Nodal support

Nodal support for all data sets was estimated using one or more of the following methods: equal-weights MP bootstrap analysis (100 pseudoreplicates, each consisting of a heuristic search using 100 random sequence addition replicates), ML bootstrap analysis (100 pseudoreplicates generated with SEQBOOT in PHYLIP and analyzed using successive approximations in PAUP*) [65,66], and Bayesian analysis (10 million generations, with the first one or two million discarded as burn-in and trees sampled every 500 generations for the *matR *and *atp*1 data sets; 10 million generations, with the first 5 million discarded as burn-in and trees sampled every 500 generations for the 3-gene data set) [61]. The GTR+Γ + I submodels used in PAUP* are not available in MrBayes; a standard GTR+Γ + I model was used for the *matR *and *atp1 *data sets instead. A partitioned model was used for the 3-gene data set (see below). Two Bayesian runs were performed for all analyses in an attempt to determine if stationarity was reached, and plots of log likelihood and parameter convergence were also evaluated; log-likelihood plots alone are insufficient for monitoring chain mixing and convergence [67,68].

#### Partitioned analyses

The 3-gene data set was also analyzed in MrBayes 3.0. A "fully partitioned" analysis was used in which the 3-gene data set was divided into seven partitions: nuclear SSU; *atpB *first, second and third codon positions; *rbcL *first, second and third codon positions. Appropriate substitution models for each data partition were chosen by computing likelihood scores for each partition on a MP tree for the 3-gene data set under 56 substitution models in PAUP* and comparing the scores in ModelTest. The GTR+Γ + I model was the best-fitting model for all partitions. The Bayesian analysis was performed with all model parameters (except branch lengths) unlinked across partitions.

#### Constraints

For the nuclear SSU rDNA data, constrained analyses were also performed. A constraint tree for 63 nonparasitic taxa was constructed using the MP topology of the "B series" tree from Soltis et al. [1] with relationships for poorly supported clades left unresolved. This tree was used as a backbone constraint for MP and ML analyses of 77 taxa including Rafflesiales. MP analyses were performed as described above. ML analyses followed a successive approximations approach similar to that described above.

#### Simulations

To investigate possible long-branch attraction in parsimony analyses of the SSU rDNA data set, two sets of simulations were performed. For the first set of simulations, a reduced data set of SSU rDNA sequences for 20 taxa (13 nonparasites and 7 Rafflesiales) was constructed and analyzed under ML (GTR+Γ + I model) in PAUP*. The tree resulting from this analysis, with its associated ML branch lengths and model parameters, was used as the model tree on which 100 data sets of length 1766 (the length of the original SSU rDNA data set) were simulated in Seq-Gen 1.2.7 [69]. For the second set of simulations, the ML tree for the full 77-taxon data set, with associated branch lengths and model parameters, was used as a model tree to simulate 100 data sets of length 1766 in Seq-Gen 1.2.7. Either MP and ML trees (20-taxon simulation) or just MP trees (77-taxon simulation) were estimated for all 100 simulated data sets. The trees (or strict consensus trees, if more than one MP or ML tree was recovered for a given simulated data set) were then inspected to determine the presence of "incorrect" clades (containing two or more "long-branch" Rafflesiales taxa) that were not present on the model tree. We do not expect to recover such clades at high frequencies unless long-branch attraction is biasing the analyses.

## List of abbreviations

Γ + I – gamma + invariant sites distribution

*atp1 *– ATP synthase alpha subunit

*atpB***– **ATP synthase beta subunit

BI – Bayesian inference

GTR – general time reversible model

HGT – horizontal gene transfer

*matK *– maturase K

*matR *– maturase R

ML – maximum likelihood

MP – maximum parsimony

*rbcL *– ribulose bisphosphate carboxylase/oxygenase, large subunit

SSU – small subunit

TBR – tree bisection-reconnection branch swapping

## Authors' contributions

DLN coordinated all aspects of the study, obtained many of the genomic DNAs, generated all the nuclear SSU rDNA, conducted the sequence alignments, and drafted the manuscript. AB conducted the majority of the mitochondrial *atp1 *and *matR *sequencing and revised the text regarding morphological character comparisons. YQ provided primers, introduced AB to the field of molecular systematics, and supervised his Ph.D thesis. RVR conducted the PCR experiments showing host contamination of *Rafflesia *DNA and generated the *matR *sequences for several taxa. FEA performed the phylogenetic analyses. All authors read and approved the final manuscript.

## Supplementary Material

Additional File 1 **MP strict consensus tree from mitochondrial *matR ***Strict consensus of 200,000+ trees obtained from maximum parsimony (unconstrained MP) analysis of the 77-taxon mitochondrial *matR *matrix. Bootstrap percentages are shown above the lines. Rafflesiales taxa are shown in bold italics. Click here for file

Additional File 2 **Strict consensus MP tree from mitochondrial *atp1 ***Strict consensus of 328 trees resulting from a MP analysis of the 71-taxon mitochondrial *atp1 *matrix. Rafflesiales taxa are shown in bold italics. Bootstrap percentages are given above the branches. Click here for file

Additional File 3 **Majority rule consensus BI tree from 3-gene data set **Majority rule consensus of 20,000 trees (10 million generations, 5 million burn-in) resulting from Bayesian analysis of the 77-taxon nuclear 3-gene matrix. Clades with Bayesian posterior probabilities are indicated above the clades. Rafflesiales taxa are shown in bold italics. Click here for file

Additional File 4 **Strict consensus constrained MP tree from nuclear SSU rDNA **Strict consensus of 6 trees resulting from the constrained MP analysis of the 77-taxon nuclear SSU rDNA matrix. Rafflesiales taxa are shown in bold italics. Bootstrap percentages are given above selected nodes (Rafflesiales). Click here for file

Additional File 5 **Taxa used in this study **MS Excel file giving taxon names and GenBank numbers for all genes used. Click here for file
